# Therapeutic Response to Secukinumab in a 36-Year-Old Woman with Hidradenitis Suppurativa

**DOI:** 10.1155/2018/8685136

**Published:** 2018-04-16

**Authors:** Astrid-Helene Ravn Jørgensen, Yiqiu Yao, Simon Francis Thomsen

**Affiliations:** ^1^Department of Dermato-Venereology and Wound Healing Centre, Bispebjerg Hospital, Copenhagen, Denmark; ^2^Department of Biomedical Sciences, University of Copenhagen, Copenhagen, Denmark

## Abstract

Hidradenitis suppurativa (HS) is a chronic inflammatory skin disorder involving primarily the intertriginous skin of the axillary, inguinal, genital, and perianal areas of the body. It is characterized by recurrent inflamed nodules and abscesses, resulting in fistulae, fibrosis, and scarring. We present a case of HS refractory to local and systemic antibiotic therapy as well as anti-TNF and anti-IL12/23 that was successfully treated with secukinumab (anti-IL17A).

## 1. Introduction

Hidradenitis suppurativa (HS) is a chronic inflammatory skin condition primarily affecting the intertriginous skin of the axillary, inguinal, genital, and perianal areas of the body. It is characterized by recurrent inflamed nodules and abscesses, resulting in fistulae, fibrosis, and scarring [1]. The prevalence of HS is thought to be approximately 1% [1].

Secukinumab (Cosentyx) is a monoclonal antibody specifically targeting IL-17A. The drug was the first anti-IL-17A biologic to be approved for the treatment of psoriasis by the Food and Drug Administration and the European Medicines Agency. Treatment with subcutaneous secukinumab (300 mg) once every week followed by once every four weeks has shown convincing results in the treatment of psoriasis [2]. Although there is no consensus on the role of interleukin-17 in the pathogenesis of HS, studies by Matusiak et al. and Kelly et al. have shown increased interleukin-17 serum levels in patients with HS and mRNA expression in lesional HS skin, respectively [3, 4].

A multinational phase II clinical trial on anti-interleukin-17 therapy in HS has been completed and two case reports suggest that secukinumab may be beneficial in HS in the short term [5, 6].

## 2. Materials and Methods

Our patient was a 36-year-old woman with Hurley II stage HS for the past 20 years with profound abscesses and fistulae affecting the axillary, perianal, and inguinal areas. She had no family history of HS. She had daily consumption of 3–8 cigarettes. She received medical treatment (citalopram) for depression and had a BMI of 30 kg/m^2^. At the first visit the patient had a Dermatology Life Quality Index (DLQI) of 17 (scale 0–30), a Hidradenitis Suppurativa Score (HSS) of 76, an overall disease bother score on a visual analogue scale (VAS) of 10 out of 10, and a physician global assessment (PGA) score of severe and an International Hidradenitis Suppurative Severity Score (IHS4) [7] of 19. CRP was increased to 20 (mg/L). All other blood samples, including liver and kidney function, leucocytes, and lipids, were normal. Previous treatments were temporary or unsuccessful and had consisted of topical clindamycin, azelaic acid, and resorcinol as well as systemic tetracycline, clindamycin plus rifampicin, isotretinoin, infliximab, adalimumab, and ustekinumab (anti-IL12/23). One abscess had been treated with surgical incision.

**Figure 1:**
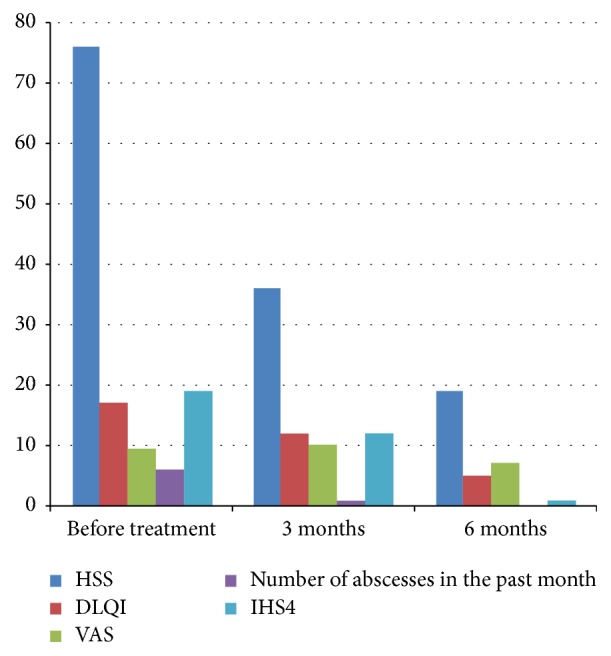
Clinical parameters during treatment of HS with secukinumab. HSS: Hidradenitis Suppurativa Score; DLQI: Dermatology Life Quality Index; VAS: visual analogue scale; IHS4: International Hidradenitis Suppurativa Severity Score System.

**Figure 2:**
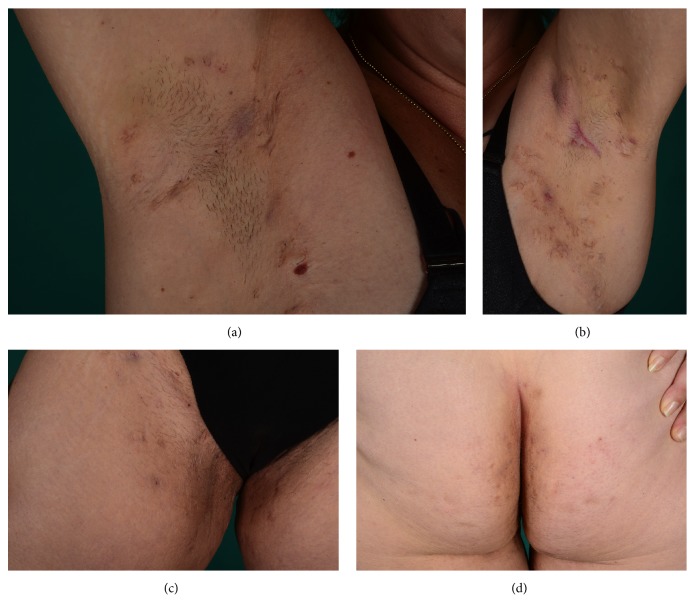
Patient photos of the axillary region and the gluteal and genitofemoral region 6 months after treatment with secukinumab showing clearance of most inflammatory lesions.

As of November 2016 we initiated treatment with 300 mg secukinumab weekly during a five-week period followed by 300 mg secukinumab monthly. After six months of treatment with secukinumab, the patient showed remarkable symptom relief with a reduction in DLQI to 5, HSS to 19, VAS to 7, and IHS4 to 1 (Figure 1). Patient photos 6 months after treatment with secukinumab showed clearance of most inflammatory lesions (Figure 2). No surgical intervention was conducted during the first 6 months of treatment with secukinumab. As of November 2017 she is still treated with secukinumab 300 mg monthly and is still in remission. During treatment the patient experienced a small relapse and a few episodes of throat infections and fever. She was treated with CO2-laser in her genital and left axillary region for two isolated lesions.

## 3. Discussion and Conclusion

Our patient showed rapid and distinct symptom relief, improved quality of life, and objective reduction in disease activity assessed on validated scales over the course of the 6-month treatment with secukinumab. Furthermore, 12 months with secukinumab and a single CO2-laser treatment provided sustained and on-going therapeutic response, which expands upon results from previous reports that have shown only short term efficacy of secukinumab in HS [5, 6].

Studies on the role of IL-17 in HS pathogenesis are lacking and widespread use of secukinumab in HS awaits evidence from future larger clinical trials.
